# Dopamine in Auditory Nuclei and Lemniscal Projections is Poised to Influence Acoustic Integration in the Inferior Colliculus

**DOI:** 10.3389/fncir.2021.624563

**Published:** 2021-03-05

**Authors:** Sharonda Harris, Renee Afram, Takashi Shimano, Bozena Fyk-Kolodziej, Paul D. Walker, Rod D. Braun, Avril Genene Holt

**Affiliations:** ^1^Department of Pharmacology and Therapeutics, University of Florida School of Medicine, Gainesville, FL, United States; ^2^Department of Ophthalmology, Visual and Anatomical Sciences, Wayne State University School of Medicine, Detroit, MI, United States; ^3^Shimano ENT Clinic, Osaka, Japan

**Keywords:** auditory system, lateral lemniscus, inferior colliculus, dopamine, tyrosine hydroxylase, immunohistochemistry, HPLC, fluorogold (FG)

## Abstract

Dopamine (DA) modulates the activity of nuclei within the ascending and descending auditory pathway. Previous studies have identified neurons and fibers in the inferior colliculus (IC) which are positively labeled for tyrosine hydroxylase (TH), a key enzyme in the synthesis of dopamine. However, the origins of the tyrosine hydroxylase positive projections to the inferior colliculus have not been fully explored. The lateral lemniscus (LL) provides a robust inhibitory projection to the inferior colliculus and plays a role in the temporal processing of sound. In the present study, immunoreactivity for tyrosine hydroxylase was examined in animals with and without 6-hydroxydopamine (6-OHDA) lesions. Lesioning, with 6-OHDA placed in the inferior colliculus, led to a significant reduction in tyrosine hydroxylase immuno-positive labeling in the lateral lemniscus and inferior colliculus. Immunolabeling for dopamine beta-hydroxylase (DBH) and phenylethanolamine N-methyltransferase (PNMT), enzymes responsible for the synthesis of norepinephrine (NE) and epinephrine (E), respectively, were evaluated. Very little immunoreactivity for DBH and no immunoreactivity for PNMT was found within the cell bodies of the dorsal, intermediate, or ventral nucleus of the lateral lemniscus. The results indicate that catecholaminergic neurons of the lateral lemniscus are likely dopaminergic and not noradrenergic or adrenergic. Next, high-pressure liquid chromatography (HPLC) analysis was used to confirm that dopamine is present in the inferior colliculus and nuclei that send projections to the inferior colliculus, including the cochlear nucleus (CN), superior olivary complex (SOC), lateral lemniscus, and auditory cortex (AC). Finally, fluorogold, a retrograde tracer, was injected into the inferior colliculus of adult rats. Each subdivision of the lateral lemniscus contained fluorogold within the somata, with the dorsal nucleus of the lateral lemniscus showing the most robust projections to the inferior colliculus. Fluorogold-tyrosine hydroxylase colocalization within the lateral lemniscus was assessed. The dorsal and intermediate nuclei neurons exhibiting similar degrees of colocalization, while neurons of the ventral nucleus had significantly fewer colocalized fluorogold-tyrosine hydroxylase labeled neurons. These results suggest that several auditory nuclei that project to the inferior colliculus contain dopamine, dopaminergic neurons in the lateral lemniscus project to the inferior colliculus and that dopaminergic neurotransmission is poised to play a pivotal role in the function of the inferior colliculus.

## Introduction

Dopamine (DA) is a neuromodulatory neurotransmitter in the brain that is involved in several processes, such as cognition, motor function, motivation, and processing of stimuli from sensory systems, including the auditory system. Dopaminergic neurotransmission has been implicated in many regions involved in processing auditory stimuli, including the inner ear, auditory brainstem, midbrain, thalamus, and cortex (Malmfors and Sachs, 1968; Jonsson, 1983; Wamsley et al., 1989; Weiner et al., 1991; Kitahama et al., 1996; Tong et al., 2005; Maison et al., 2012). Alterations in DA neurotransmission within the central auditory pathway have also been associated with auditory stimulation as well as noise-induced damage and hearing loss (Tong et al., 2005; Fyk-Kolodziej et al., 2015; Wu et al., 2020).

One critical brain region within the central auditory system where evidence for dopaminergic input has been found is the inferior colliculus (IC; Tong et al., 2005; Gittelman et al., 2013; Fyk-Kolodziej et al., 2015; Nevue et al., 2016a,b). The IC is a midbrain nucleus that is a major site of integration for ascending and descending input within the auditory system. Previous studies have reported on the production of tyrosine hydroxylase (TH), the rate-limiting enzyme for DA synthesis, within the IC (Paloff and Usunoff, 2000; Holt et al., 2005) with many TH immunolabeled terminals negative for dopamine β-hydroxylase (DBH) or phenylethanolamine N-methyltransferase (PNMT), enzymes that are absent in catecholaminergic neurons that produce DA (Fyk-Kolodziej et al., 2015). DA receptors have also been identified in this brain region (Wamsley et al., 1989; Weiner et al., 1991; Fyk-Kolodziej et al., 2015). Moreover, DA has been found to modulate the activity of IC neurons in response to auditory stimuli (Gittelman et al., 2013), and DA receptor modulation has been shown to alter auditory-stimulated IC responses within a behavioral context (Satake et al., 2012; Muthuraju et al., 2014). These studies suggest that DA in the IC modulates the processing of auditory stimuli. However, the origin(s) of DA within the IC remain unclear.

Recent reports suggest that the subparafascicular thalamic nucleus (SPF) may account for some of the DA in the IC (Nevue et al., 2016a,b; Batton et al., 2019; Hoyt et al., 2019). The IC also receives input from several nuclei within the central auditory pathway that has been implicated in DA neurotransmission (Campbell et al., 1987; Cransac et al., 1996; Gabriele et al., 2000; Behrens et al., 2002; Mulders and Robertson, 2005), including the cochlear nucleus (CN), lateral lemniscus (LL), and superior olivary complex (SOC), as well as descending input from the auditory cortex (AC). DA was previously identified, using high-pressure liquid chromatography (HPLC) analysis, in the CN (Cransac et al., 1996). Besides, immunoreactivity for TH was found within the lateral superior olive, suggesting that DA in the IC could also come from this projection region (Mulders and Robertson, 2004). Another brain region with projections to the IC that has also been suggested to contain DA is the LL (Holt et al., 2005; Tong et al., 2005).

Our previous study found that neurons in the LL primarily produce TH in the absence of dopamine β-hydroxylase (DBH) or PNMT, enzymes needed, in addition to TH, for the synthesis of norepinephrine (NE) and epinephrine (E), respectively (Tong et al., 2005). This finding provided additional evidence in support of dopaminergic neurons within the LL. The LL consists of three subregions, the dorsal nucleus (DNLL), the intermediate nucleus (INLL), and the ventral nucleus (VNLL), each of which projects to the IC (Glendenning et al., 1981; Covey and Casseday, 1991; Schofield and Cant, 1997; Kelly et al., 1998). TH labeling was previously found to be most prominent within the VNLL and INLL (Tong et al., 2005). The reports of DA and TH throughout the auditory pathway increase the likelihood that other brain regions, in addition to the recently identified SPF, contribute to DA neurotransmission within the IC. Although numerous studies provide support for the presence of DA in the IC, to date, neither evidence for a functional pool of DA capable of neurotransmission or a comparison of DA levels across auditory nuclei have been reported.

Therefore, the present study uses 6-hydroxydopamine (6-OHDA) lesioning and HPLC analysis to conclusively establish the presence of DA available for neurotransmission within the IC and other auditory nuclei that project to the IC. Also, a putative source of DA from the LL to the IC was examined by combining tract-tracing and immunolabeling for enzymes in the catecholaminergic synthesis pathway. The majority of TH immunolabeled neurons appear to be dopaminergic since there was sparse to no immunolabeling for DBH or PNMT in LL cell bodies. Co-labeled (FG-TH) neurons projecting to the IC from the LL were identified, demonstrating that projections from the LL are a likely source of DA to the IC.

## Materials and Methods

### Subjects

Specific pathogen-free adult male Sprague–Dawley rats (Charles River Laboratories, Wilmington, MA, USA) were used (*n* = 38) following guidelines for animal care issued by the National Institute of Health and following the Institutional Animal Care and Use Committee at Wayne State University.

### Stereotaxic Surgery

Naïve rats (*n* = 10) were anesthetized with a mixture of xylazine (8 mg/kg of 20 mg/ml i.m.) and ketamine (75 mg/kg of 100 mg/ml i.m.) with their body temperature maintained using a water circulating heating pad. The head of each animal was positioned in a stereotaxic frame and once the skull was exposed, a craniotomy was made just above the IC using rat specific stereotaxic coordinates (rostrocaudal 0.876 mm from bregma, 0.18 mm medio-lateral, 0.32 mm dorso-ventral from pia) obtained from a rat brain atlas (Paxinos and Watson, 2014). Either 0.5 μl of FG (4% dissolved in H_2_O; FluoroChrome Inc., Engelwood, CO, USA), 6-OHDA (20 μg), a dopaminergic neurotoxin, or vehicle (normal saline) was pressure injected bilaterally into the IC using a 5 μl Hamilton fine needle syringe over two minutes. The injection targeted the mid-rostro-caudal region of the IC. The syringe was allowed to remain in place for five additional minutes after the injection was complete. Dental cement (Durelon, Dental Products, St. Paul, MN, USA) was used to seal the craniotomy and the skin incision was closed using non-continuous sutures (Ethicon, Somerville, NJ, USA). Following surgeries, the animals were administered 3 ml of warm (60°C) sterile 0.9% sodium chloride solution subcutaneously and allowed to recover under a heating lamp. The animals were subsequently placed in their cages and monitored until normal activity resumed. They were then returned to the animal facility and allowed to recover from surgeries for 7 days at which time their sutures were removed.

### HPLC-ED Analysis

Naïve rats (*n* = 20) with no prior surgical exposure were used for HPLC analysis coupled to electrochemical detection (HPLC-ED). The rats were decapitated, brains collected and individual samples from the CN, SOC, LL, IC, and AC were dissected, quickly frozen in liquid nitrogen, and stored at −80° C until analyzed for DA levels. Entire brain regions of interest were dissected by hand either as a single unit (CN, IC, AC) or by punch (LL and SOC). Nuclei were collected bilaterally and were weighed and analyzed for HPLC-ED. Ultimately, results from bilateral samples from individual subjects were averaged. Cryostat sections from the remaining brain (after dissection) was used to confirm that the brain regions of interest were removed (data not shown). Just before analysis, each sample was weighed again and homogenized in 300 μl ice-cold 0.1 M perchloric acid containing 1.0% ethanol, 0.02% EDTA, and centrifuged (16,000 *g*) for 30 min at 4°C. The supernatant was taken for measurement of DA content. The HPLC system consisted of a Waters WISP autoinjector, a Waters 510 dual piston pump, an external pulse dampener (Rainin), Waters Guard Pak column, and a C-18 (100 × 3.2 mm, 5 μm packing, Perkin Elmer) column. The mobile phase contained 0.75 mM sodium phosphate, 0.5 mM EDTA, 1.4 mM octane sulfonic acid, and 7% acetonitrile (final pH 3.0). A coulometric detector (Model 5011, ESA) configured with three electrodes was used. An ESA model 5020 guard cell (+400 mV) was placed before the WISP injector and an ESA model 5011 analytical cell (first electrode at −40 mV; second electrode at +350 mV) was placed immediately after the column. The signal from the second analytical electrode was recorded and analyzed by a Waters baseline 810 Chromatography Workstation via a Waters Interface Module. The signal produced by the oxidation of DA was measured and compared with that from known standard concentrations. The data are expressed as nanogram DA per milligram of tissue weight (median ± S.E.M.). Statistical analysis of HPLC data was performed with one-way ANOVA and Tukey’s test for *post hoc* comparisons using GraphPad Prism version 8.0 (GraphPad Prism software, San Diego, CA, USA). *p* < 0.05 was considered significant.

### Fixation and Tissue Processing for Immunohistochemistry (IHC)

The 10 rats that underwent stereotaxic delivery of 6-OHDA or FG were maintained for 7 days following injections to allow 6-OHDA induced lesioning of TH neurons or retrograde transport of the FG from the terminals within the IC to the cell bodies of projection neurons. The rats were then administered 0.22 ml/kg of Fatal-Plus (Vortech Pharmaceutical, Dearborn, MI, USA) i.p. and perfused transcardially with 9.25% sucrose in 0.1 M phosphate buffer (PB) pH 6.8 followed by 4.0% paraformaldehyde in 0.1 M PB using an automated perfusion system (myNeuroLab, St. Louis, MO, USA). After perfusion, the brains were dissected and post-fixed in the same fixative for 1 h at room temperature and then cryoprotected at 4°C in 30% sucrose. Brains were then coronally sectioned throughout their entire rostrocaudal extent into 30 μm sections using a freezing microtome. The sections were mounted in serial order on Histo-Bond slides (Thermo Fisher Scientific, Pittsburgh, PA, USA) and stored at −80°C until processed.

### Immunohistochemistry (IHC)

Immunolabeling was performed in sections containing the IC as well as the subregions of the LL (DNLL, INLL, and VNLL) using secondary antibodies conjugated with a fluorophore or biotinylated for visualization. Additional rats (*n* = 8) were also used for MAP2/TUJ1, DBH, and PNMT immunolabeling of the LL.

### Primary Antibodies

• *TH*; rabbit (AB152); Chemicon, Temecula, CA, USA; 1:500 (fluorescence) and 1:1,000 (DAB).
• *MAP2*; mouse (MAB3418); Chemicon, Temecula, CA, USA; 1:200.
• *TUJ1*; mouse (AB2728521); Covance, Berkeley, CA, USA: 1:200.
• *DBH*; mouse (MAB308); Millipore, Temecula, CA, USA; 1:4,000.
• *PNMT*; sheep (ab146); Chemicon, Temecula, CA, USA; 1:2,000.

Sections were rinsed three times for 5 min each in 0.1 M phosphate-buffered saline (PBS) with 0.3% Triton X-100 (PBST) and pre-incubated for 3 h at room temperature in blocking solution consisting of 3% normal serum in PBST from the species corresponding with the appropriate secondary antibody used. Each primary antibody was diluted in PBS with 1% normal serum and sections were incubated in primary antibody for 48–72 h at 4°C. The sections were then rinsed again three times in PBS before incubation in secondary antibodies.

For fluorescent immunolabeling, the sections were incubated in species-specific secondary antibodies conjugated to Cy3 (red fluorescence) for 2 h at room temperature. Alexa 568 or Cy3-conjugated secondary antibodies (Jackson ImmunoResearch Laboratories, West Grove, PA, USA) were used at a dilution of 1:1,000. The sections then were rinsed several times in PBS and coverslipped using Vectashield Mounting Medium (Vector Laboratories, Burlingame, CA, USA).

For immunoperoxidase immunolabeling of 6-OHDA and saline-treated animals, the sections were incubated with biotinylated secondary antibodies (Vector Labs) for 2 h at room temperature. Sections from the LL and IC of 6-OHDA and saline-treated animals were processed together. The sections were then incubated in an avidin-biotin complex (Vectastain Elite Kit, Vector Labs). The avidin-biotin/HRP complex was visualized using 0.0125% diaminobenzidine (Millipore Sigma, St. Louis, MO, USA) in PBS and 0.075% H_2_O_2_ and allowed to develop for six minutes before the reaction was stopped by rinsing the sections with double-distilled H_2_O. The sections were then air-dried, dehydrated, and coverslipped.

## Image and Data Analysis

Sections were visualized using a Leica DM4500 fluorescence microscope equipped with appropriate fluorescent filters. Images were taken with a Photometrics Coolsnap EZ, 12 bit, 20 MHz monochrome digital camera (Mager Scientific, Dexter, MI, USA). First, to determine the total area of each of the LL subregions to be used for cell counting and data analysis, specific guidelines were applied. The number of FG- and TH-immunolabeled cell bodies (both single and co-labeled) were counted and compared across the rostral, middle, and caudal regions of each of the LL subregions. The specific guidelines used to establish the total area for cell counting were based on anatomical landmarks and applied as follows:

### Rostral Region

The total area of the DNLL in the rostral region is 700 μm × 800 μm and the total area of the INLL is 700 μ m × 700 μm. A 600 μm × 600 μm area for both the DNLL and INLL was used for cell counting. For the DNLL, the counted area was located 150 μm from the lateral edge and 100 μm ventral to the cerebral aqueduct (CA). For the INLL, the counted area was positioned 150 μm from the lateral edge and 700 μm ventral to the CA. The total area of the VNLL is 400 μm × 1,100 μm, therefore the VNLL was split into two separate counting areas of 400 μm × 400 μm. The counted areas were located 150 μm from the lateral edge and 1,300 μm ventral to the CA.

### Middle Region

The total area of the DNLL in the middle region is 600 μm × 600 μm. A 500 μm × 500 μm area that was 150 μm from the lateral edge and 200 μm dorsal to the CA was used for cell counting. The total area of the INLL in the middle region is 400 μm × 500 μm. A 400 μm × 400 μm counted area located 150 μm from the lateral edge and 300 μm ventral to the CA was used. The total area of the VNLL is 400 μm × 1,100 μm, which was split into two counted areas of 300 μm × 400 μm. These areas were located 150 μm lateral to the edge and 800 μm ventral to the CA.

### Caudal Region

The total area of the caudal region of the DNLL is 800 μm × 700 μm. The counted area of 500 μm × 600 μm was 150 μm from the lateral edge and 300 μm dorsal to the CA. The total area of the INLL is 600 μm × 600 μm. The counted area of 500 μm × 500 μm was 150 μm from the lateral edge and 500 μm ventral to the CA. The total area of the VNLL is 400 μm × 1,000 μm, which was split into two counted regions of 300 × 400 μm. These areas were located 150 μm from the lateral edge and 1,400 μm ventral to the CA.

Each grid square equaled 100 μm^2^. Cells that fell on the grid lines were not counted, and any cells outside of the specific counting areas were also not counted. Photographs of the same region were taken using different fluorescent filters and were overlaid to determine the number of cells that colocalized FG and TH. Statview (Version 5.0.1, SAS Institute, Cary, NC, USA) was used for statistical analysis of the images and generation of graphs. *p* < 0.05 was considered significant.

## Results

### TH Immunoreactivity in the LL and IC Is Reduced by Lesioning With 6-OHDA

TH immunoreactivity was evaluated in the IC and LL of both non-lesioned animals and animals lesioned after delivery of 6-OHDA to the IC. The neurotoxin, 6-OHDA, causes selective degeneration of cells producing TH, such as noradrenergic and dopaminergic neurons, through uptake by dopamine transporters. Following lesioning, TH immunoreactivity was examined in the IC and LL of lesioned animals and compared to that of non-lesioned animals.

### Non-lesioned Animals

**Figure 1:**
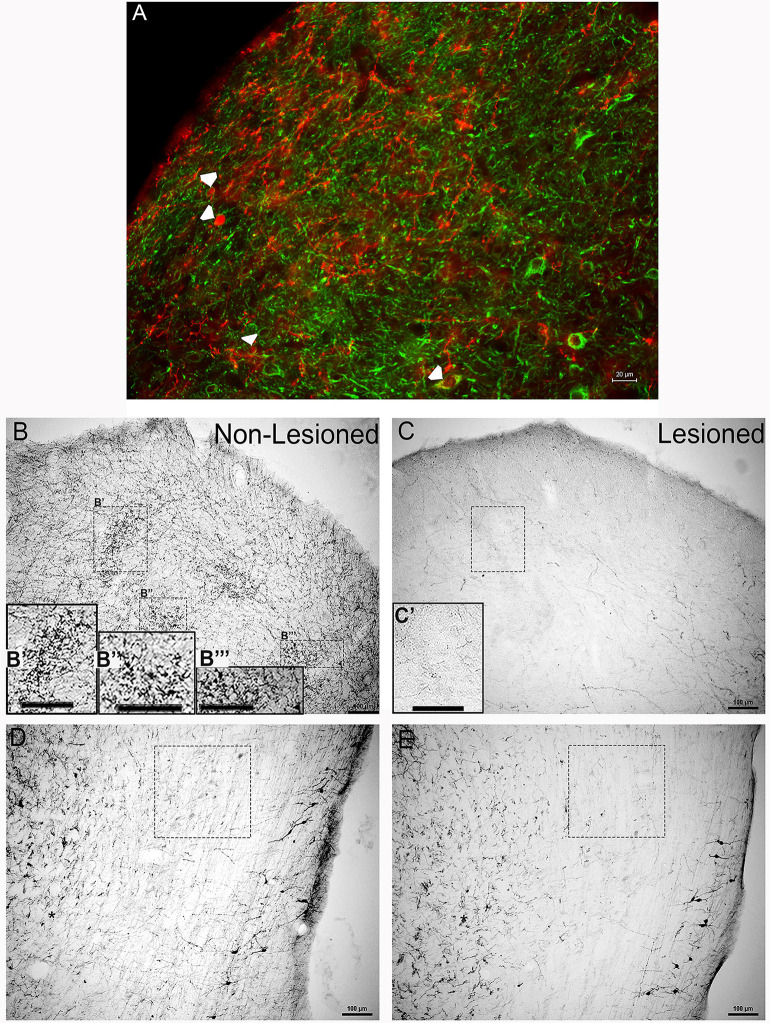
Tyrosine hydroxylase (TH) immunoreactivity in the inferior colliculus (IC) and lateral lemniscus (LL) before and after neurotoxic lesioning with 6-OHDA. **(A)** Immuno-fluorescent labeling of TH (red) and the neuronal markers microtubule-protein2 and tubulin beta III (MAP2/TUJ1, green) in the IC before lesioning. Arrowheads represent neuronal somata double-labeled for TH and MAP2/TUJ1. **(B,B′,B”,B”′)** Clusters of TH positive immunolabeled somas, dendrites, varicosities, and en passant boutons were present in the IC before lesioning. **(C,C′)** The neurotoxin 6-OHDA was delivered to the IC, with TH immunolabeling performed after lesioning. **(D)** TH positive immunolabeling was present in the LL before 6-OHDA delivery into the IC. **(E)** Immunolabeling for TH in the LL was also performed after 6-OHDA lesioning. Panels **(B′,B”,B”′,C′)** are enlargements of insets in **(B,C)** respectively, **(B′,B”’,C′)**. The boxes in **(D,E)** represent TH labeling of somata and neuropil, before and after lesioning, in comparable regions of the LL with varying degrees of en passant boutons and varicosities. The scale bars = 20 μm for **(A)** and 100 μm for **(B–E)** and **(B′,B”,B”′)**.

Similar to our previous studies (Tong et al., 2005; Fyk-Kolodziej et al., 2015), TH immunoreactive cell bodies, dendrites and terminals were found in both the IC (Figures 1B,C) and LL (Figures 1D,E). Triple-labeling of TH with the neuronal markers, MAP2 (microtubule-protein 2) and TUJ1 (tubulin beta III) verified that TH labeling is localized to neurons in the IC (Figure 1A). Patches of TH labeling containing somas, but primarily dendrites, and varicosities, were observed throughout the mid-rostrocaudal IC (Figure 1B,B′,B”,B”′). Labeling resembling terminal fields was homogenously dispersed throughout the nucleus. In the LL, somas, dendrites, and en passant boutons were labeled for TH (Figure 1D). At the lateral edges of the tissue, heavily labeled varicosities and somata with numerous highly branched dendrites were observed (Figure 1D).

### 6-OHDA Lesioned Animals

Delivery of the neurotoxin, 6-OHDA, into the IC resulted in significantly reduced TH immunoreactivity (Figures 1C,E). In the IC, there was an obvious loss of the TH positive patches, with somata, dendrites, and varicosities as well as en passant boutons throughout the neuropil (Figure 1C,C′). As for the LL, there was a reduction in TH immunoreactivity that manifest as reduced labeling of somas, en passant boutons, and varicosities in the neuropil (Figure 1E). However, at the lateral edges of the tissue, while the dendritic processes were less branched, TH immunoreactivity in these somata appeared to be mostly spared (Figure 1E).

Overall, these findings provide the support that TH positive cells and fibers found in the IC were reduced by 6-OHDA lesioning in the IC, and this lesioning led to a reduction in TH positive projections from the LL to the IC.

### TH Immunoreactivity in the LL Results Primarily From Dopaminergic Neurons

**Figure 2:**
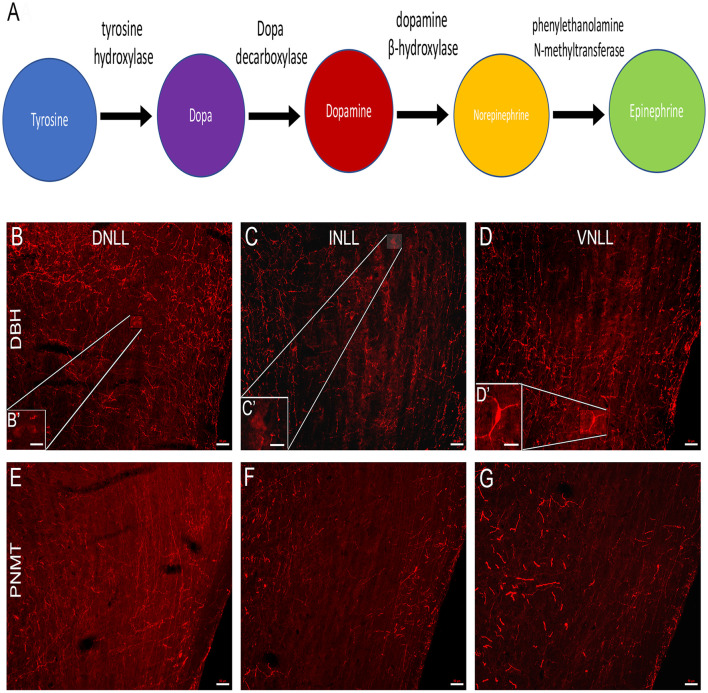
Immunolabeling of norepinephrine (NE) and epinephrine (E) cell bodies in the LL. **(A)** The catecholaminergic biosynthetic pathway for DA, NE, and E begins with the conversion of the amino acid tyrosine to L-Dopa by the rate-limiting enzyme, TH. L-Dopa is then converted to dopamine by the enzyme L-dopa decarboxylase. NE is formed from DA by the enzyme dopamine β-hydroxylase (DBH) and E is synthesized from NE by the enzyme phenylethanolamine-N-methyltransferase (PNMT). **(B–D)** Each subdivision of the LL was analyzed for the presence of DBH and PNMT positive cells, indicative of NE and E cells, respectively. **(B,B′)** Labeling of DBH positive cells in the dorsal nucleus (DNLL), **(C,C′)** intermediate nucleus (INLL), and **(D,D′)** ventral nucleus (VNLL) of the LL. Labeling of PNMT positive cells in the **(E)** DNLL, **(F)** INLL, and **(G)** VNLL of the LL. Panels **(B′–D′)** are an enlarged view of the insets in **(B–D)**, respectively. The scale bars = 50 μm for **(B–G)** and 20 μm for **(B’–D’)**.

Although the reduction in TH immunoreactivity in the LL and IC following 6-OHDA lesioning suggests that dopaminergic somata and terminals are likely to be present in the LL and IC, the nature of the catecholaminergic neurons within the LL was examined further, given that 6-OHDA can lesion both noradrenergic and dopaminergic neurons. Therefore, immunoreactivity for the enzymes involved in the biosynthesis of norepinephrine (NE) and epinephrine (E) was assessed. Figure 2A represents the catecholamine biosynthetic pathway. TH is the rate-limiting enzyme for the synthesis of each of the catecholamines. DA is produced from tyrosine by the enzyme TH. NE is produced from DA by the enzyme dopamine beta-hydroxylase (DBH) and NE is converted to E by the enzyme PNMT. To build on our previous studies demonstrating the presence of both DA and NE in LL neurons (Tong et al., 2005; Fyk-Kolodziej et al., 2015) immunolabeling for DBH and PNMT was used to determine whether the LL neurons produce NE or E (Figures 2B–G).

### DBH Immunoreactivity in the LL

Sparse DBH labeling of cell bodies (~2%) was observed within the DNLL. There were, however, DBH-labeled varicosities present (Figure 2B) with dense labeling dorsally. In the INLL, there was a similar level of DBH immunolabeling of cell bodies. The density of varicosities appeared to be less than that observed in the DNLL. The bouton-like varicosities in the INLL were evenly dispersed (Figure 2C). The VNLL showed the lowest numbers of DBH immuno-positive somata when compared to the DNLL and the INLL (Figure 2D). The DBH positive fibers were more strongly labeled than the somas in each of the LL subregions (Figures 2B–D). These data suggest that very few NE producing neurons are present in the LL.

### PNMT Immunoreactivity in the LL

There were no immuno-positive PNMT cell bodies found in in the DNLL (Figure 2E), INLL (Figure 2F), or VNLL (Figure 2G). There were, however, numerous PNMT-immunolabeled varicosities found in each of the LL subdivisions. Thus, the data show a lack of E-expressing neurons in the LL. Overall, these data provide evidence that the catecholaminergic neurons within the LL are dopaminergic.

### Nuclei Within the Central Auditory Pathway Contain DA

**Figure 3:**
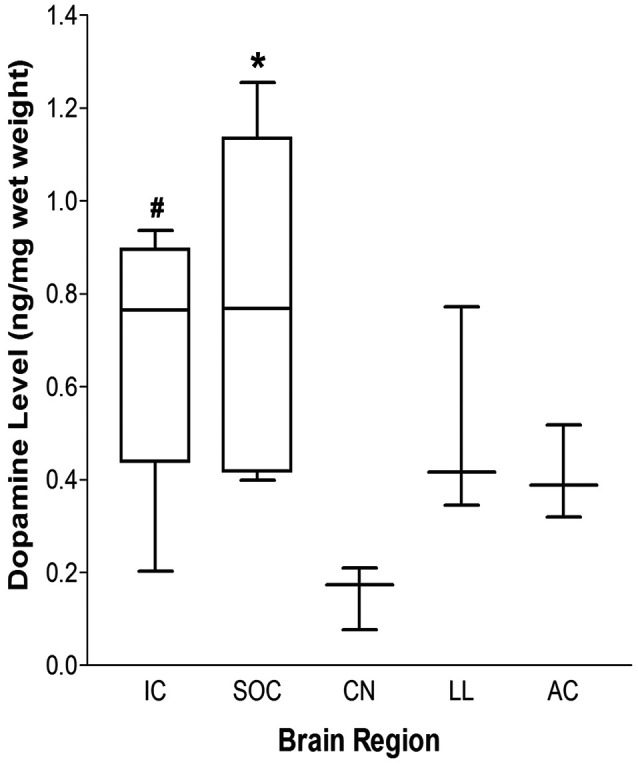
Dopamine (DA) levels within auditory nuclei of the central auditory pathway. DA levels within central auditory nuclei were determined by HPLC and measured as ng/mg tissue weight (median ± S.E.M.). ^#^*p* < 0.05 IC vs. CN; *p < 0.01 SOC vs. CN. IC: *n* = 6; SOC: *n* = 8; LL, AC and CN: *n* = 4.

Using HPLC-ED, DA levels were evaluated in several auditory-related nuclei and reported based on tissue weight (ng DA/mg tissue weight; Figure 3). DA was detected in each of the regions examined [IC: 0.699 ± 0.107 (*n* = 6); SOC: 0.787 ± 0.137 (*n* = 8); LL: 0.487 ± 0.99 (*n* = 4); AC: 0.403 ± 0.44 (*n* = 4); CN: 0.158 ± 0.029 (*n* = 4); median ± S.E.M. ng/mg tissue weight]. The highest DA levels were found in the IC and SOC. The level of DA in the SOC was significantly higher than that in the CN (one-way ANOVA: *p* = 0.010; Tukey’s *post hoc* test: SOC vs. CN, *p* < 0.01). Similarly, DA levels in the IC were also significantly higher than the levels in the CN (one-way ANOVA; *p* = 0.010; Tukey’s *post hoc* test: IC vs. CN, *p* < 0.05). The levels of DA in the LL and AC were comparable and less than those in the IC and SOC, although the differences were not statistically significant (Figure 3). These data confirm the presence of DA in the IC and LL, as well as other important nuclei within the central auditory system.

### Administration of FG Into the IC Combined With TH Labeling in the LL Demonstrate Dopaminergic Projections From the LL to the IC

**Figure 4:**
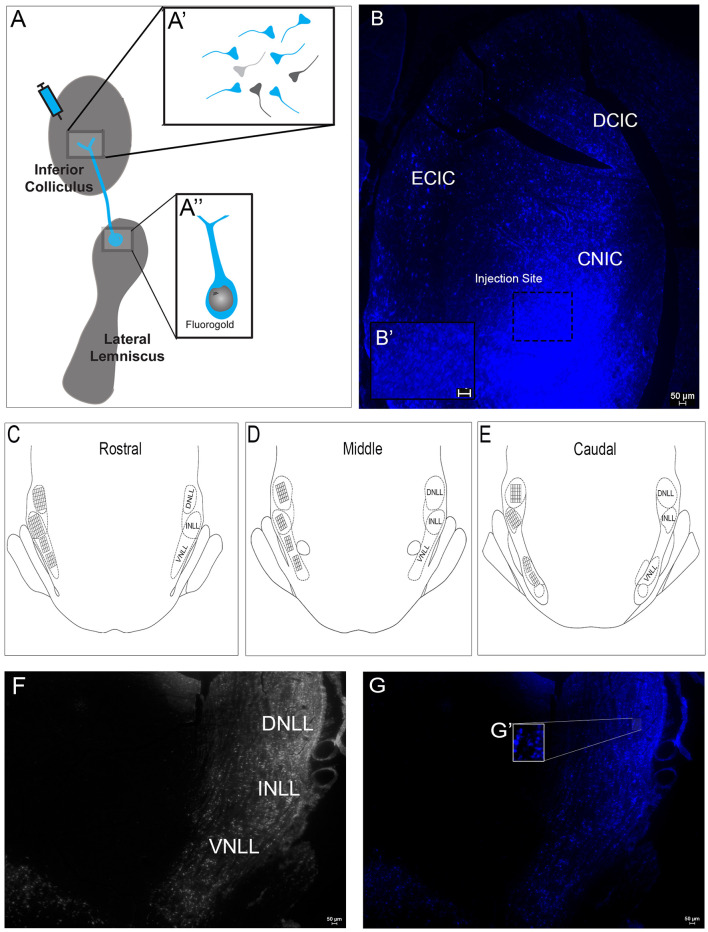
Fluorogold (FG) labeling within the subregions of the LL following injection of FG in the IC. **(A)** A representative schematic of the FG delivery to the IC. FG was taken up by IC axonal terminals and transported to cell bodies in the LL. **(A’)** A schematic of axon terminals in the IC following FG delivery. Some terminals take up FG with retrograde transport to the LL (blue). Other terminals take up FG but are from neurons that project from regions other than LL (dark gray). Some terminals may not take up FG (light gray). **(A”)** A schematic showing an FG-labeled soma with dendrites in the LL after FG delivery to the IC. **(B)** Deposition of FG was targeted to the mid-rostro-caudal region of the IC. **(B’)** The inset shows extra-synaptic and -somatic FG in the neuropil. The 0.5 μl FG solution appeared to spread ~240 μm both rostrally and caudally and primarily involved the central nucleus with occasional involvement of the dorsal cortex and external cortex in some animals. **(C–E)** A schematic representation of the placement of the reticle grids used for counting cells throughout the rostral **(C)**, middle **(D)** and caudal **(E)** extent of the dorsal nucleus (DNLL), intermediate nucleus (INLL) and ventral nucleus (VNLL) of the LL. Each square within a grid represents 100 μm^2^. **(F)** Representative image of the subregions of the LL—DNLL, INLL, and VNLL. **(G,G’)** Fluorescent image of **(F)** showing FG in somata throughout the LL after delivery into the IC. **(G’)** An enlargement of the inset in **(G)** showing LL somata containing FG. Scale bars = 50 μm.

Retrograde labeling with FG was used to examine projections to the IC from the LL (Figure 4A). FG was injected into the IC (Figure 4B,B′). The spread of the FG was apparent in sections ~240 μm rostrally and caudally from the point of delivery. At the injection site, the small volume of FG was primarily contained within the central nucleus of the IC (Figure 4B), where cellular and extracellular FG was observed (Figure 4B′). However, there was occasional spread into the dorsal and external cortex in some animals. Intracollicular FG transport was observed both ipsilateral and contralateral to the injection site (Figure 4B). To characterize the extent of the projection from the LL to the IC and to determine whether the pathway contains dopamine, FG containing and TH-labeled neurons in the LL were investigated. The cell counting method employed used strictly defined parameters to determine the area within each LL subregion to be counted and thus allowed precise identification of FG positive only, TH positive only, and FG + TH positive cells (Figures 4C–E). Somata containing FG were found throughout each of the subregions of the LL (DNLL, INLL, and VNLL; Figures 4F,G,G′). These results are in line with numerous previous studies that show that the LL sends major projections to the IC.

### Colocalization of FG and TH Indicates Dopaminergic Projections From the DNLL to the IC

**Figure 5:**
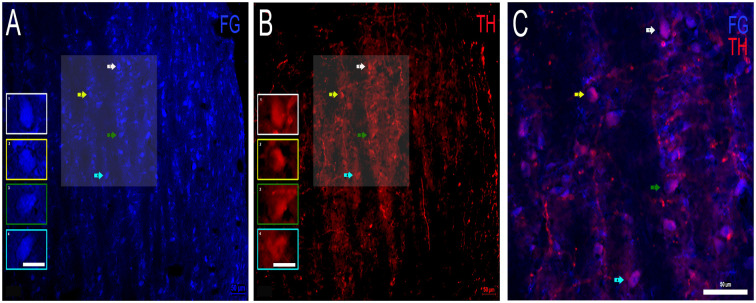
Colocalization of FG and TH within the DNLL of the LL. **(A)** FG containing cell bodies (blue) and **(B)** TH labeled cell bodies (red) in the DNLL. Insets in **(A,B)** represent individual labeled cells of interest. Panel **(C)** is an expanded overlay of the shaded area of interest in **(A,B)** and represent FG and TH colocalized cells (pink). Scale bars = 50 μm **(A–C)** and 25 μm for images within the individual insets.

First, FG containing and TH positive somata were examined separately in the DNLL. Cell bodies containing FG were found throughout the DNLL (Figure 5A). TH labeling of cell bodies was also found throughout the DNLL (Figure 5B), although the TH immunoreactivity was not as robust as the FG transport. TH positive fibers were also present. When sections for FG and TH were analyzed for colocalization (Figure 5C), numerous DNLL somata were found to co-localize FG and TH. The TH immunoreactivity could be visualized within FG containing somata but did not completely fill the neurons (Figure 5C). The data show that a proportion of the neurons within the DNLL that send projections to the IC are dopaminergic.

### Dopaminergic Projections From the INLL to the IC

**Figure 6:**
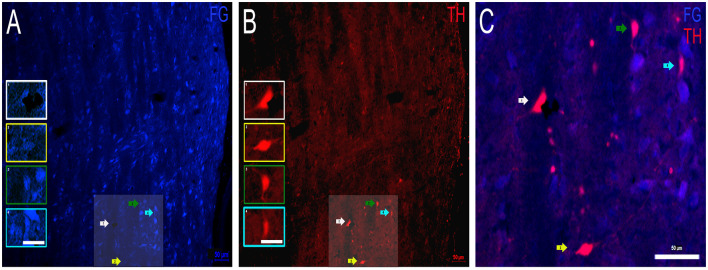
Colocalization of FG and TH within the INLL of the LL. **(A)** FG containing cell bodies (blue) and **(B)** TH labeled cell bodies (red) in the INLL. Insets in **(A,B)** represent individual cells of interest. Panel **(C)** is an expanded overlay of the shaded area of interest in **(A,B)** and represent FG and TH colocalized cells (pink). Scale bars = 50 μm **(A–C)** and 25 μm for images within the individual insets.

Next, FG containing and TH labeled INLL somata were examined (Figure 6). As in the DNLL, numerous cell bodies in the INLL were positive for FG (Figure 6A). As for TH immunostaining, TH-positive cell bodies and fibers were also present (Figure 6B). The intensity of the FG within the somata was greater than the intensity of the TH labeling. However, there were patches of intensely labeled TH somata located throughout the mid-rostrocaudal region of the INLL (Figure 6B). There were also TH-positive fibers found throughout the INLL. When FG containing neurons and TH immunoreactivity were analyzed for co-localization, numerous somata were found to colocalize FG and TH (Figure 6C). As with the DNLL, these data demonstrate that the INLL also provides dopaminergic projections to the IC.

### Dopaminergic Projections From the VNLL to the IC

**Figure 7:**
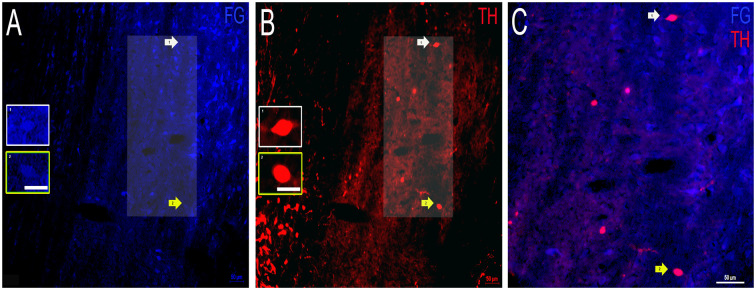
Colocalization of FG and TH within the VNLL of the LL. **(A)** FG containing cell bodies (blue) and **(B)** TH labeled cell bodies (red) in the VNLL. Insets in **(A,B)** represent individual cells of interest. Panel **(C)** is an expanded overlay of the area of interest in **(A,B)** and represent FG and TH colocalized cells (pink). Scale bars = 50 μm **(A–C)** and 25 μm for images within the individual insets.

Finally, FG containing and TH labeled somata were examined in the VNLL. As for FG labeling, there were cell bodies in the VNLL that contain FG, although to a lesser extent than FG in the DNLL or INLL (Figure 7A). Both somata and fibers labeled for TH and were also identified. However, the TH labeled somata and fibers were noticeably fewer when compared to the DNLL and the INLL (Figure 7B). Colocalization of FG and TH was also found in the VNLL, but both the intensity of the transported FG and TH immunolabeling was less compared to other LL subdivisions (Figure 7C). These data provide evidence that a proportion of neurons within the VNLL projecting to the IC are dopaminergic, albeit a smaller proportion of cells than those found in the DNLL or INLL.

### Quantitative Analysis Shows Regional Differences in LL Dopaminergic Projections to the IC

**Figure 8:**
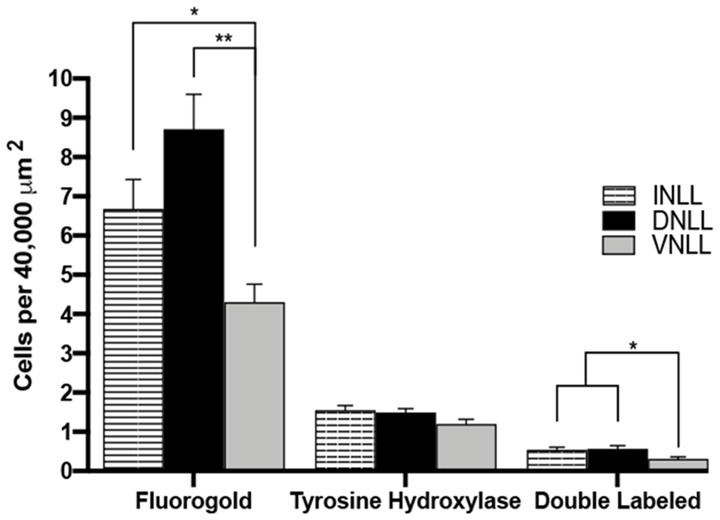
Quantification of FG and TH colocalization within the subregions of the LL. FG, TH and colocalized FG and TH labeled cells in the DNLL, INLL, and VNLL subregions of the LL. Error bars indicate standard error. *P < 0.05, **P < 0.01.

The number of FG and TH colocalized cells were quantified and represented in Figure 8. The number of FG containing cells per 40,000 mm^2^ were DNLL: 8.71 ± 0.89; INLL: 6.68 ± 0.75 and VNLL 4.0 ± 0.46 (mean ± S.E.M). There was a higher number of FG positive cell bodies in the DNLL and INLL compared to the VNLL (*post hoc* Scheffe Test: INLL vs. VNLL, *P* < 0.05; DNLL vs. VNLL, *P* < 0.001; DNLL vs. INLL, *P* > 0.05). There was a similar number of TH positive cell bodies in each of the subregions of the LL (DNLL: 1.49 ± 0.10; INLL: 1.55 ± 0.12; VNLL: 1.20 ± 0.12 TH labeled cells/40,000 mm^2^). However, the number of TH labeled cell bodies was about 30% of the number of FG containing cell bodies. Double-labeling for FG and TH was also found within each of the subregions of the LL and represented about 30% of the total TH positive cells (DNLL: 0.57 ± 0.08; INLL: 0.54 ± 0.07; VNLL: 0.31 ± 0.05 FG + TH double-labeled cells/40,000 mm^2^). As with FG labeling, colocalization of FG and TH was greater in the DNLL and INLL, although there were nearly 50% fewer co-labeled cells found in the VNLL (Scheffe Test: DNLL vs. VNLL, *P* < 0.05; INLL vs. VNLL, *P* < 0.05; DNLL vs. INLL, *P* > 0.05).

The rostral to the caudal extent of FG, TH, and double-labeled cells were also characterized (data not shown). The rostral, middle, and caudal regions contained roughly equal numbers of FG-labeled, TH-labeled or FG-TH colocalized cells. Overall, cells colocalized for FG and TH were found throughout each of the subregions of the LL, with the most prominent double-labeling found in the DNLL and INLL. The results indicate that a subpopulation of dopaminergic neurons located throughout the LL project to the IC.

## Discussion

### The IC Receives Direct Dopaminergic Projections From the LL

Previous studies have suggested that the IC receives dopaminergic projections. While there is substantial indirect evidence for the presence of DA in the IC, direct evidence of DA content within this nucleus is lacking. The present study demonstrated DA content in the IC and identified the LL as a source of DA to the IC. HPLC analysis revealed DA throughout the central auditory pathway, with the highest levels found in the SOC and IC. Given the previous findings that the LL contains cells immuno-positive for TH (Tong et al., 2005), the present report, using tract-tracing studies with fluorogold and lesioning studies, further characterized TH immunoreactivity within the LL and its projections to the IC.

The presence of dopaminergic neurons in the LL and IC was supported by findings in which TH positive neurons were first verified in the IC by triple immunolabeling of TH and the neuronal markers, MAP2 and TUJ1, and then by lesioning with 6-OHDA, a neurotoxin that specifically targets TH-expressing cells. The 6-OHDA was injected specifically into the IC and the results showed a significant reduction in TH immunolabeling within IC cell bodies after 6-OHDA lesioning, as well as a reduction in TH-labeled varicosities.

As for the LL, the reduction in TH immunoreactivity within terminal fields was dramatic. The extent and complexity of dendritic processes appeared reduced in lesioned animals, but the TH-labeled somata were spared. The extent and progression of 6-OHDA induced degeneration of dopaminergic neurons is highly influenced by the site of the injection (Agid et al., 1973; Przedbroski et al., 1995; Przedbroski and Tieu, 2006). The dopamine transporter regulates spatial and temporal aspects of dopaminergic neurotransmission by governing the synaptic reuptake of DA. The DA transporter is also the method by which 6-OHDA is taken into dopaminergic neurons (Blum et al., 2001; Vaughan and Foster, 2013). Although 6-OHDA lesioning of IC neurons led to a significant reduction in TH labeling in the LL, the reduction was partial. Possibly the lack of reduction in TH positive cells bodies in the LL was because TH immunostaining studies were performed one week after 6-OHDA lesioning. Previous studies have shown that 6-OHDA lesioning led to significantly less degeneration of DA cell bodies in the substantia nigra compared to degeneration of dopamine terminals in the striatum two weeks following the lesion (Grant and Clarke, 2002). Also, axon terminals are more sensitive to 6-OHDA neurodegeneration than axons or somata (Malmfors and Sachs, 1968; Jonsson, 1983). Therefore, more time after 6-OHDA lesioning may have been needed to allow for the complete reduction in TH immunoreactivity within LL cell bodies. There was also a reduction in TH immunoreactivity within fibers found in the LL. Given that 6-OHDA was injected into the IC, the reduction in fiber labeling within the LL indicates that the LL provides a source of TH positive projections to the IC. However, since there was also a reduction in TH positive terminals within the LL, the IC may send TH positive projections to the LL. These data point to the likelihood that the LL provides a source of dopamine to the IC. Consequently, future studies are needed to determine the extent to which the LL sends dopaminergic projections to auditory nuclei other than the IC.

The present results corroborate our previous study showing that the LL contains TH immunoreactive neurons, with very few somata immunoreactive for DBH and none immunoreactive for PNMT, indicative of NE and E neurons, respectively (Tong et al., 2005). These findings provide strong support for the conclusion that the TH positive projections from the LL to the IC are primarily dopaminergic. The DNLL likely provides limited noradrenergic input to the IC. Consistent with this finding, DBH positive cell bodies have been previously reported in the LL (Wynne and Robertson, 1996). Conversely, DBH and PNMT positive fibers, characterized by en passant swellings, were found throughout the LL. There was a substantive population of fibers within the LL that were immunolabeled for DBH or PNMT, suggesting that other brain regions likely send both noradrenergic and adrenergic projections to the LL. One possibility is that NE may activate TH neurons in the LL and provide sympathetic modulation of auditory information integration in the IC, though this hypothesis needs further examination in future studies. The immunohistochemical technique used in the present study did not distinguish whether these fibers are from axons, axonal terminals, or dendrites; therefore, the use of electron microscopy is needed to address these important questions.

Tract tracing studies using FG and TH immunohistochemistry were examined separately and then in combination to determine the degree of FG and TH colocalization of neurons in the LL. Extensive FG labeling was found throughout each of the subregions of the LL (DNLL, INLL, and VNLL), in line with numerous published studies demonstrating major LL projections to the IC (Kudo, 1981; Druga and Syka, 1984; Kelly et al., 1998). TH immunoreactivity was found in LL cell bodies in each of the subregions examined. Interestingly, there were differences in the intensity of TH immunoreactivity within the subregions of the LL. The INLL and VNLL contained TH positive cells that were more intensely labeled compared to those in the DNLL, although the number of TH positive cells was higher in the DNLL and INLL compared to the VNLL. These results are similar to previous studies reporting differences in the intensity of TH labeling within auditory nuclei (Tong et al., 2005; Fyk-Kolodziej et al., 2015), particularly in the subregions of the LL. The DNLL and INLL primarily contain GABAergic and glutamatergic neurons respectively, whereas the VNLL contains neurons that co-label for GABA and glycine (Zhang et al., 1998; Ito and Oliver, 2010; Moore and Trussell, 2017). Our previous study identified TH terminals that colocalized with glycinergic and glutamatergic terminals in the IC, as well as TH positive terminals that occasionally co-localized with GABAergic terminals (Fyk-Kolodziej et al., 2015). Consequently, the previous results of colocalization of TH with glutamate or TH with glycine in the IC combined with our current results, specifically implicate the INLL and VNLL, respectively, as possible sources of DA in the IC. Some dopaminergic neurons within the LL may not co-localize with other neurotransmitters and could represent a separate population of neurons, that project to the IC or other auditory nuclei. On the other hand, a significant number of TH positive cells within each of the subregions of the LL did not colocalize with FG, suggesting that the LL sends dopaminergic projections to regions beyond the IC. However, further studies focused on the neurotransmitter phenotype and projection of TH-positive lemniscal neurons are needed to characterize dopaminergic neurons in the LL.

Overall, these results provide substantial evidence for LL projections to the IC, as has been previously established, and expand our previously published findings to show that the LL provides a source of DA to the IC.

### DA Within the IC

Several previous studies have suggested the presence of DA within the IC. For example, DA receptors were identified in the IC (Wamsley et al., 1989; Weiner et al., 1991), and DA delivered to the IC was found to alter spontaneous and acoustically-induced neuronal activity (Gittelman et al., 2013). However, none of these reports directly measured DA levels in the IC as was done in this study. The use of HPLC in the present study enabled the selective and sensitive measurement of DA within discrete brain regions, unlike methods such as immunohistochemistry and western blot analysis, which only provide information about the localization or amount of protein that is associated with DA. Thus, assessment of DA in auditory nuclei has previously been indirect. Moreover, previous studies have shown that there are subsets of TH positive neurons that can synthesize DA, but do not possess the molecular machinery to accumulate and/or store DA (Weihe et al., 2006; Morales and Root, 2014). The ability to store DA is particularly important since free cytosolic dopamine is quickly degraded. Detection of DA using HPLC confirms that TH positive neurons in the auditory nuclei produce and store DA that is available for neurotransmission. Therefore, HPLC analysis provides convincing evidence that DA is, indeed, present in the IC and LL. Besides, DA was also found in the CN, SOC, and AC, auditory nuclei that are also involved in the processing of auditory information and known to send projections to the IC. However, one limitation of HPLC is that the nature of DA content, i.e., whether the source is somatic or synaptic, cannot be differentiated. Nonetheless, the present study has confirmed the presence of DA available for neurotransmission within the IC, as well as in the LL and other auditory nuclei that project to the IC.

The subparafascicular thalamic nucleus (SPF), a forebrain region that contains dopamine neurons within the A11 dopaminergic cell group, has been reported to send dopaminergic projections to the IC (Takada et al., 1988; Nevue et al., 2016a,b). In one study, the SPF and auditory nuclei that project to the IC (e.g., LL) were examined using FG and TH colocalization, but only the SPF was reported to have TH immunoreactive somata (Nevue et al., 2016a). Another recent study reported that activation of SPF neurons induced DA release in the IC (Batton et al., 2019). While these reports suggest the SPF as the primary source of DA to the IC, the present study provides evidence that the LL is a source of DA to the IC. Since one-third of the TH positive cells in the LL were found to project to the IC the discrepancy between the current study and that of Nevue et al., is unclear. However, the site of the fluorogold injection or the TH antibody used may be contributors. The present study also used a clearly defined and rigorous method (Figures 4C–E) of identifying and counting cells positive for FG and TH which likely enabled a more complete assessment of TH immunoreactivity in the LL. Finally, the aforementioned published studies were performed in mice and not in rats as in the present study and thus, species differences may have led to the differential findings.

The nucleus sagulum, a midbrain tegmental region, lies lateral to the DNLL and sends projections to the cortex of the IC (Brunso-Bechtold et al., 1981; Henkel and Shneiderman, 1988; Hutson et al., 1991; Beneyto et al., 1998; Motts and Schofield, 2009; Schofield et al., 2014). Previous studies report choline acetyltransferase, GABA, and glycine as neurotransmitters produced by sagulum neurons (Beneyto et al., 1998; Motts and Schofield, 2009). Given that darkly labeled TH-positive neurons were also present at the lateral borders of the DNLL and INLL, possibly at least some of those neurons were part of the nucleus sagulum. Several of the neurons found near the lateral edge of the tissue also contained FG. Since the sagulum cannot be easily distinguished in the current study, future investigation is needed to clarify the precise catecholaminergic composition and localization of potential TH labeled sagulum neurons.

The presence of DA in the IC was also previously suggested using fast-scan cyclic and pair pulse voltammetry (Batton et al., 2019). Using voltammetry, DA cannot be easily distinguished from NE. DA was determined indirectly, using DA and NE transporter inhibitors and thus, DA could not be conclusively confirmed as in the current study (Batton et al., 2019). The present report provides clear evidence of a previously unreported dopaminergic pathway from the LL to IC.

### Function of DA in the IC

The role of DA within the IC has not been fully elucidated. DA has been found to modulate IC neuronal activity and the response of IC neurons to auditory stimuli (Gittelman et al., 2013). DA applied to the IC produced heterogeneous effects on the activity of IC neurons, resulting in both an increase and decrease in spontaneous and sound-stimulated neuronal firing in the IC (Gittelman et al., 2013). Additionally, inhibition of DA receptor activation within the IC by a DA D2 receptor antagonist has been found to modulate auditory-evoked defensive behavior, such as unconditioned fear (De Oliveira et al., 2014; Muthuraju et al., 2014). A D2 agonist in the IC reduced pre-pulse inhibition of the acoustic startle response (Satake et al., 2012). The ascending input from several auditory nuclei, including the LL, also modulates IC neurons and the response of IC neurons to auditory stimuli (Kidd and Kelly, 1996; Batra and Fitzpatrick, 2002; Wang et al., 2018). The subregions of the LL have differential effects on auditory information processing in the IC. Past studies have shown that the DNLL and its connections to the IC are involved in binaural processing and sound localization (Li and Kelly, 1992; Ito et al., 1996; Kidd and Kelly, 1996; Kelly et al., 1998; van Adel et al., 1999), whereas the VNLL is thought to process temporal features of sounds and shape-selective responses to sounds of different durations (Covey and Casseday, 1991; Batra and Fitzpatrick, 2002). The LL may, therefore, contribute some of its neuromodulatory effects on auditory processing within the IC through dopaminergic neurotransmission and thus impact IC modulation of ascending and descending auditory pathways.

### Future Directions

The current findings validate previous studies that establish the presence of DA in the CN, SOC, LL, IC, and AC and provide evidence for the LL as a source of DA in the IC. Our HPLC findings support the presence of endogenous DA in these auditory nuclei. Interestingly, DA neurons within the SPF do not contain the dopamine transporter (Koblinger et al., 2014). Given the importance of the dopamine transporter in maintaining tissue levels of DA, it is possible that HPLC analysis of DA levels in SPF-projecting regions could be underestimated (Vaughan and Foster, 2013). Therefore, an examination of dopamine transporter immunolabeling in the IC and LL is an important area of future inquiry. Additional studies would also expand our understanding of DA neurotransmission in the LL-IC pathway. Dopamine can either activate (D1-type receptors) or inhibit (D2-type) the activity of neurons. Dual-labeled immunohistochemistry for DA receptor subtypes and TH combined with tract tracing studies could be used to examine the subtype of dopaminergic receptors present on IC neurons that receive projections from the LL. Moreover, deafness leads to a reduction in TH labeling in the LL and IC (Tong et al., 2005). Another important future study would be to investigate whether hearing loss alters DA levels in auditory nuclei and leads to alterations in the dopaminergic LL projections to the IC. The findings from these studies would provide knowledge of the functional role of the dopaminergic LL to the IC pathway under normal conditions as well as during impaired hearing and hearing loss.

## Conclusion

The present study provides considerable evidence that several nuclei within the central auditory system contain a source of DA capable of dopaminergic neurotransmission. This study also confirms that the LL sends dopaminergic projections to the IC. TH labeled terminals in the IC have been shown to co-localize with glutamate and glycine terminals and associate closely with GABA terminals. Thus, LL dopaminergic projections to the IC are well-positioned to modulate glutamate, GABA, and glycine neurotransmission in the IC and ascending auditory neurotransmission. Elucidation of DA’s function in the IC and the dopaminergic LL-IC pathway will likely further current knowledge of auditory information processing in the ascending and descending auditory pathway. Moreover, a more thorough understanding of this pathway may provide new molecular and therapeutic targets to aid in the identification and prevention of hearing loss due to injury, and, after exposure to loud noise or blast (e.g., in urban settings or in the military).

## Data Availability Statement

The original contributions presented in the study are included in the article, further inquiries can be directed to the corresponding author.

## Ethics Statement

The animal study was reviewed and approved by the Institutional Animal Care and Use Committee at Wayne State University.

## Author Contributions

SH: formal analysis, data curation, and writing—draft. RA: methodology, data curation, and writing—original draft. TS: methodology, visualization, data curation, and writing—original draft. BF-K: methodology, visualization, data curation, writing—review, and editing. PW: methodology, validation, resources, data curation, writing—review, and editing. RB: data curation, writing—review, and editing. AH: conceptualization, methodology, validation, data curation, writing—original draft, supervision, project management, and funding acquisition. All authors contributed to the article and approved the submitted version.

## Conflict of Interest

The authors declare that the research was conducted in the absence of any commercial or financial relationships that could be construed as a potential conflict of interest.

## Abbreviations

6-OHDA: 6-hydroxydopamine
AC: auditory cortex
CA: cerebral aqueduct
CN: cochlear nucleus
DA: dopamine
DBH: dopamine-β-hydroxylase
DNLL: dorsal nucleus lateral lemniscus
FG: fluorogold
HPLC: high-pressure liquid chromatography
IC: inferior colliculus
IHC: immunohistochemistry
INLL: intermediate nucleus lateral lemniscus
LL: lateral lemniscus
NDS: normal donkey serum
PB: phosphate buffer
PBS: phosphate-buffered saline
PBST: phosphate-buffered saline with 0.3% Triton X-100
PNMT: phenyl ethanolamine-*N*-methyl transferase
SOC: superior olivary complex
TH: tyrosine hydroxylase
VNLL: ventral nucleus lateral lemniscus.

## References

[B1] Agid Y. Javoy F. Glowinski J. Bouvet D. Sotelo C. (1973). Injection of 6-hydroxydopamine into the substantia nigra of the rat. II. Diffusion and specificity. Brain Res. 58, 291–301. 10.1016/0006-8993(73)90002-4 4756131

[B2] Batra R. Fitzpatrick D. C. (2002). Processing of interaural temporal disparities in the medial division of the ventral nucleus of the lateral lemniscus. J. Neurophysiol. 88, 666–675. 10.1152/jn.2002.88.2.666 12163520

[B3] Batton A. D. Blaha C. D. Bieber A. Lee K. H. Boschen S. L. (2019). Stimulation of the subparafascicular thalamic nucleus modulates dopamine release in the inferior colliculus of rats. Synapse 6:e22073. 10.1002/syn.22073 30291737

[B4] Behrens E. G. Schofield B. R. Thompson A. M. (2002). Aminergic projections to cochlear nucleus *via* descending auditory pathways. Brain Res. 955, 34–44. 10.1016/s0006-8993(02)03351-6 12419519

[B5] Beneyto M. Winer J. A. Larue D. T. Prieto J. J. (1998). Auditory connections and neurochemistry of the sagulum. J. Comp. Neurol. 401, 329–351. 10.1002/(sici)1096-9861(19981123)401:3<329::aid-cne3>3.0.co;2-w 9811112

[B6] Blum D. Torch S. Lambeng N. Nissou M. F. Benabid A. L. Sadoul R.. (2001). Molecular pathways involved in the neurotoxicity of 6-OHDA, dopamine and MPTP: contribution to the apoptotic theory in Parkinson’s disease. Prog. Neurobiol. 65, 135–172. 10.1016/s0301-0082(01)00003-x 11403877

[B7] Brunso-Bechtold J. K. Thompson G. C. Masterton R. B. (1981). HRP study of the organization of auditory afferents ascending to central nucleus of inferior colliculus in cat. J. Comp. Neurol. 197, 705–722. 10.1002/cne.901970410 7229134

[B8] Campbell M. J. Lewis D. A. Foote S. L. Morrison J. H. (1987). Distribution of choline acetyltransferase-, serotonin-, dopamine-β-hydroxylase-, tyrosine hydroxylase-immunoreactive fibers in monkey primary auditory cortex. J. Comp. Neurol. 261, 209–220. 10.1002/cne.902610204 2887595

[B9] Covey E. Casseday J. H. (1991). The monaural nuclei of the lateral lemniscus in an echolocating bat: parallel pathways for analyzing temporal features of sound. J. Neurosci. 11, 3456–3470. 10.1523/JNEUROSCI.11-11-03456.1991 1941092 PMC6575535

[B10] Cransac H. Cottet-Emard J. M. Pequignot J. M. Peyrin L. (1996). Monoamines (norepinephrine, dopamine, serotonin) in the rat medial vestibular nucleus: endogenous levels and turnover. J. Neural Transm. 103, 391–401. 10.1007/BF01276416 9617784

[B11] De Oliveira A. R. Colombo A. C. Muthuraju S. Almada R. C. Brandão M. L. (2014). Dopamine D2-like receptors modulate unconditioned fear: role of the inferior colliculus. PLoS One 9:e104228. 10.1371/journal.pone.0104228 25133693 PMC4136794

[B12] Druga R. Syka J. (1984). Ascending and descending projections to the inferior colliculus in the rat. Physiol. Bohemoslov. 45, 247–252. 6709726

[B13] Fyk-Kolodziej B. E. Shimano T. Gafoor D. Mirza N. Griffith R. D. Gong T. W.. (2015). Dopamine in the auditory brainstem and midbrain: co-localization with amino acid neurotransmitters and gene expression following cochlear trauma. Front. Neuroanat. 9:88. 10.3389/fnana.2015.00088 26257610 PMC4510424

[B14] Gabriele M. L. Brunso-Bechtold J. K. Henkel C. K. (2000). Development of afferent patterns in the inferior colliculus of the rat: projection from the dorsal nucleus of the lateral lemniscus. J. Comp. Neurol. 20, 6939–6949. 10.1523/JNEUROSCI.20-18-06939.2000 10602095

[B15] Gittelman J. X. Perkel D. J. Portfors C. V. (2013). Dopamine modulates auditory responses in the inferior colliculus in a heterogeneous manner. J. Assoc. Res. Otolaryngol. 14, 719–729. 10.1007/s10162-013-0405-0 23835945 PMC3767870

[B16] Glendenning K. K. Brusno-Bechtold J. K. Thompson G. C. Masterton R. B. (1981). Ascending auditory afferents to the nuclei of the lateral leminscus. J. Comp. Neurol. 197, 673–703. 10.1002/cne.901970409 7229133

[B17] Grant R. J. Clarke P. B. S. (2002). Susceptibility of ascending dopamine projections to 6-hydroxydopamine in rats: effect of hypothermia. Neuroscience 115, 1281–1294. 10.1016/s0306-4522(02)00385-8 12453497

[B18] Henkel C. K. Shneiderman A. (1988). Nucleus sagulum: projections of a lateral tegmental area to the inferior colliculus in the cat. J. Comp. Neurol. 271, 577–588. 10.1002/cne.902710408 2454973

[B520] Holt A. G. Asako M. Lomax C. A. MacDonald J. W. Tong L. Lomax M. I.. (2005). Deafness-related plasticity in the inferior colliculus: gene expression profiling following removal of peripheral activity. J. Neurochem. 93, 1069–1086. 10.1111/j.1471-4159.2005.03090.x 15934929

[B19] Hoyt J. M. Perkel D. J. Portfors C. V. (2019). Dopamine acts *via* D2-like receptors to modulate auditory responses in the inferior colliculus. eNeuro 6:ENEURO.0350–19.2019. 10.1523/ENEURO.0350-19.2019 31548368 PMC6791829

[B20] Hutson K. A. Glendenning K. K. Masterton R. B. (1991). Acoustic chiasm. IV: eight midbrain decussations of the auditory system in the cat. J. Comp. Neurol. 312, 105–131. 10.1002/cne.903120109 1720792

[B22] Ito T. Oliver D. L. (2010). Origins of glutamatergic terminals in the inferior colliculus identified by retrograde transport and expression of VGLUT1 and VGLUT2 genes. Front. Neuroanat. 4:135. 10.3389/fnana.2010.00135 21048892 PMC2967334

[B21] Ito M. van Adel B. Kelly J. B. (1996). Sound localization after transection of the commissure of Probst in the albino rat. J. Neurophysiol. 76, 3493–3502. 10.1152/jn.1996.76.5.3493 8930288

[B23] Jonsson G. (1983). “Chemical lesioning techniques: monoamine neurotoxins,” in Handbook of Chemical Neuroanatomy, eds Björklund A. Hökfelt T. (Amsterdam: Elseiver), 463–507.

[B24] Kelly J. B. Liscum A. Van Adel B. Ito M. (1998). Projections from the superior olive and lateral lemniscus to tonotopic regions of the rat’s inferior colliculus. Hear. Res. 116, 43–54. 10.1016/s0378-5955(97)00195-0 9508027

[B25] Kidd S. A. Kelly J. B. (1996). Contribution of the dorsal nucleus of the lateral lemniscus to binaural responses in the inferior colliculus of the rat: interaural time delays. J. Neurosci. 16, 7390–7397. 10.1523/JNEUROSCI.16-22-07390.1996 8929445 PMC6578946

[B26] Kitahama K. Sakamoto N. Jouvet A. Nagatsu I. Pearson J. (1996). Dopamine-β-hydroxylase and tyrosine hydroxylase immunoreactive neurons in the human brainstem. J. Chem. Neuroanat. 10, 137–146. 10.1016/0891-0618(96)00111-1 8783042

[B27] Koblinger K. Füzesi T. Ejdrygiewicz J. Krajacic A. Bains J. S. Whelan P. J. (2014). Characterization of A11 neurons projecting to the spinal cord of mice. PLoS One 9:e109636. 10.1371/journal.pone.0109636 25343491 PMC4208762

[B28] Kudo M. (1981). Projections of the nuclei of the lateral lemniscus in the cat: an autoradiographic study. Brain Res. 221, 57–69. 10.1016/0006-8993(81)91063-5 6168337

[B29] Li L. Kelly J. B. (1992). Inhibitory influence of the dorsal nucleus of the lateral lemniscus on binaural responses in the rat’s inferior colliculus. J. Neurosci. 12, 4530–4539. 10.1523/JNEUROSCI.12-11-04530.1992 1432109 PMC6576013

[B30] Maison S. F. Liu X.-P. Eatock R. A. Sibley D. R. Grandy D. K. Charles Liberman M. (2012). Dopaminergic signaling in the cochlea: receptor expression patterns and deletion phenotypes. J. Neurosci. 32, 344–355. 10.1523/JNEUROSCI.4720-11.2012 22219295 PMC3313790

[B31] Malmfors T. Sachs C. (1968). Degeneration of adrenergic nerves produced by 6-hydroxydopamine. Eur. J. Pharmacol. 3, 89–92. 10.1016/0014-2999(68)90056-3 5654676

[B32] Moore L. A. Trussell L. O. (2017). Corelease of inhibitory neurotransmitters in the mouse auditory midbrain. J. Neurosci. 37, 9453–9464. 10.1523/JNEUROSCI.1125-17.2017 28847813 PMC5618263

[B33] Morales M. Root D. H. (2014). Glutamate neurons within the midbrain dopamine regions. Neuroscience 282, 60–68. 10.1016/j.neuroscience.2014.05.032 24875175 PMC4397110

[B34] Motts S. D. Schofield B. R. (2009). Sources of cholinergic input to the inferior colliculus. Neuroscience 160, 103–114. 10.1016/j.neuroscience.2009.02.036 19281878 PMC2700879

[B35] Mulders W. H. A. M. Robertson D. (2004). Dopaminergic olivocochlear neurons originate in the high frequency region of the lateral superior olive of guinea pigs. Hear. Res. 187, 122–130. 10.1016/s0378-5955(03)00308-3 14698093

[B36] Mulders W. H. A. M. Robertson D. (2005). Catecholaminergic innervation of guinea pig superior olivary complex. J. Chem. Neuroanat. 30, 230–242. 10.1016/j.jchemneu.2005.09.005 16236480

[B37] Muthuraju S. Nobre M. J. Saito V. M. N. Brandao M. L. (2014). Distinct effects of haloperidol in the mediation of conditioned fear in the mesolimbic system and processing of unconditioned aversive information in the inferior colliculus. Neuroscience 261, 195–206. 10.1016/j.neuroscience.2013.11.063 24384225

[B38] Nevue A. A. Elde C. J. Perkei D. J. Portfors C. V. (2016a). Dopaminergic input to the inferior colliculus in mice. Front. Neuroanat. 9:168. 10.3389/fnana.2015.00168 26834578 PMC4720752

[B39] Nevue A. A. Felix R. A. Portfors C. V. (2016b). Dopaminergic projections of the subparafascicular thalamic nucleus to the auditory brainstem. Hear. Res. 341, 202–209. 10.1016/j.heares.2016.09.001 27620513 PMC5111623

[B4800] Paloff A. M. Usunoff K. G. (2000). Tyrosine hydroxylase-like immunoreactive synaptic boutons in the inferior colliculus of the cat. Ann. Anat. 182, 423–426. 10.1016/S0940-9602(00)80047-3 11035636

[B480] Paxinos G. Watson C. (2014). The Rat Brain, in Stereotaxic Coordinates. 7th Edn. San Diego, CA: Academic Press.

[B41] Przedbroski S. Leviver M. Jiang H. Ferreira M. Jackson-Lewis V. Donaldson D.. (1995). Dose-dependent lesions of the dopaminergic nigrostriatal pathway induced by instrastriatal injection of 6-hydroxydopamine. Neuroscience 67, 631–647. 10.1016/0306-4522(95)00066-r 7675192

[B40] Przedbroski S. Tieu K. (2006). “Toxic animal models,” in Neurodegenerative Diseases, ed Beal M. F. (Cambridge, MA: Cambridge University Press), 196–221.

[B42] Satake S. Yamada K. Melo L. L. Barbosa Silva R. (2012). Effects of microinjections of apomorphine and haloperidol into the inferior colliculus on prepulse inhibition of the acoustic startle reflex in rat. Neurosci. Lett. 509, 60–63. 10.1016/j.neulet.2011.12.052 22230886

[B43] Schofield B. R. Cant N. B. (1997). Ventral nucleus of the lateral lemniscus in guinea pigs: cytoarchitecture and inputs from the cochlear nucleus. J. Comp. Neurol. 379, 363–385. 10.1002/(sici)1096-9861(19970317)379:3<363::aid-cne4>3.0.co;2-1 9067830

[B44] Schofield B. R. Mellott J. G. Motts S. D. (2014). Subcollicular projections to the auditory thalamus and collateral projections to the inferior colliculus. Front. Neuroanat. 8:70. 10.3389/fnana.2014.00070 25100950 PMC4103406

[B45] Takada M. Li Z. K. Hattori T. (1988). Single thalamic dopaminergic neurons project to both the neocortex and spinal cord. Brain Res. 455, 346–352. 10.1016/0006-8993(88)90093-5 2900059

[B46] Tong L. Altschuler R. A. Holt A. G. (2005). Tyrosine hydroxylase in rat auditory midbrain: distribution and changes following deafness. Hear. Res. 206, 28–41. 10.1016/j.heares.2005.03.006 16080996

[B47] van Adel B. A. Kidd S. A. Kelly J. B. (1999). Contribution of the commissure of Probst to binaural evoked responses in the rat’s inferior colliculus: interaural time differences. Hear. Res. 130, 115–130. 10.1016/s0378-5955(98)00226-3 10320103

[B48] Vaughan R. A. Foster J. D. (2013). Mechanisms of dopamine transporter regulation in normal and disease states. Trends Pharmacol. Sci. 34, 489–496. 10.1016/j.tips.2013.07.005 23968642 PMC3831354

[B49] Wamsley J. K. Gehlert D. R. Filloux F. M. Dawson T. M. (1989). Comparison of the distribution of D-1 and D-2 dopamine receptors in the rat brain. J. Chem. Neuroanat. 2, 119–137. 2528968

[B50] Wang H. Shen S. Zheng T. Bi L. Li B. Wang X.. (2018). The role of the dorsal nucleus of the lateral lemniscus in shaping the auditory response properties of the central nucleus of the inferior collicular neurons in the albino mouse. Neuroscience 390, 30–45. 10.1016/j.neuroscience.2018.08.015 30144510

[B51] Weihe E. Depboylu C. Schütz B. Schäfer M. K. H. Eiden L. E. (2006). Three types of tyrosine hydroxylase-positive CNS neurons distinguished by dopa decarboxylase and VMAT2 co-expression. Cell. Mol. Neurobiol. 26, 659–678. 10.1007/s10571-006-9053-9 16741673 PMC4183211

[B52] Weiner D. M. Levey A. I. Sunahara R. K. Niznik H. B. O’Dowd B. F. Seeman P.. (1991). D1 and D2 dopamine receptor mRNA in rat brain. Proc. Natl. Acad. Sci. U S A 88, 1859–1863. 10.1073/pnas.88.5.1859 1825729 PMC51125

[B53] Wu J. S. Yi E. Manca M. Javaid H. Lauer A. M. Glowatzki E. (2020). Sound exposure dynamically induces dopamine synthesis in cholinergic LOC efferents for feedback to auditory nerve fibers. eLife 9:e52419. 10.7554/eLife.52419 31975688 PMC7043886

[B54] Wynne B. Robertson D. (1996). Localization of dopamine-β-hydroxylase-like immunoreactivity in the superior olivary complex of the rat. Audiol. Neurootol. 1, 54–64. 9390790

[B55] Zhang D. X. Li L. Kelly J. B. Wu S. H. (1998). GABAergic projections from the lateral lemniscus to the inferior colliculus of the rat. Hear. Res. 117, 1–12. 10.1016/s0378-5955(97)00202-5 9557973

